# An analytical approximation to the AdEx neuron model allows fast fitting to physiological data

**DOI:** 10.1186/1471-2202-12-S1-P81

**Published:** 2011-07-18

**Authors:** Loreen Hertäg, Joachim Haß, Tatiana Golovko, Daniel Durstewitz

**Affiliations:** 1 Bernstein-Center for Computational Neuroscience Heidelberg-Mannheim, Central Institute of Mental Health, Heidelberg University, Mannheim, 68159, Germany

## 

Fitting spiking neuron models to physiological data sets is often a tedious, time-consuming business involving several ad-hoc assumptions. At one end of the spectrum, biophysically detailed models can often reproduce electrophysiological data to arbitrary degree. However, because of the large number of parameters, this very laborious process runs into the risk of serious over-fitting: Different parameter configurations may give similarly good fits to a set of ‘training data’ [1], but it is not clear how these different models would perform on test data that has not been used for parameter tuning. At the other end of the spectrum, analytical solutions for leaky-integrate-&-fire (LIF) models would allow a fast fitting process, but their intrinsic dynamics is too poor to reproduce the spiking behaviour of real neurons to a reasonable degree. For many purposes, however, it is important to have simple, yet in some sense still physiologically realistic neuron models. Recent extensions of the basic LIF neuron model [2-4] have made progress into this direction by providing simple models which are able to reproduce voltage traces and spiking dynamics of real neurons to an astonishing degree. Most of these studies used voltage traces to fit the model, which are obtained by somatic injections of fluctuating current inputs into neurons recorded from acute brain slices. Moreover, some of these models [2,4] also perform quite well on sections of these recordings that are different from the data used for training. However, as both training and test data consist of voltage traces that are generated by input currents with the same distribution, this approach come at the price that the considered range of inputs is restricted. Hence, the model may not generalize to different input regimes.

In this work, we extend this approach along two directions: First, rather than fitting model parameters to fluctuating voltage traces, we use in-vitro recorded f/I (spike rate over current) curves of cortical layer-5 pyramidal cells in mice, covering the whole range of spike rates up to the point of depolarization block. In this way, the adaptive exponential LIF model (AdEx) [5] is fitted to perfectly match the training set consisting of the f/I curve, and then tested on recordings obtained under fluctuating current input as in previous approaches [2,4]. Second, we derive an analytical approximation to the model [6] which yields a closed expression for the f/I curve. This approach is based on the separation of time scales [6], assuming that the time constant of the adaptive current is much slower than the membrane time constant. Thus, the fitting process can be performed analytically and exactly, which is about one order of magnitude faster than fits produced via numerical integration. The approximated model still produces a remarkably low prediction error on the distinct test set consisting of fluctuating current inputs. Thus, we have created a neuron model which can be adjusted to physiological f/I curves very quickly, which is physiologically realistic in the sense that it generalizes well to independent test sets, and is also mathematically tractable.
